# Early subacute frontal callosal microstructure and language outcomes after stroke

**DOI:** 10.1093/braincomms/fcae370

**Published:** 2025-01-21

**Authors:** Veronika Vadinova, Sonia L E Brownsett, Kimberley L Garden, Tracy Roxbury, Katherine O’Brien, David A Copland, Katie L McMahon, Aleksi J Sihvonen

**Affiliations:** Queensland Aphasia Research Centre, University of Queensland, Brisbane 4029, Australia; School of Health and Rehabilitation Sciences, University of Queensland, Brisbane 4072, Australia; NHMRC Centre for Research Excellence in Aphasia Recovery & Rehabilitation, La Trobe University, Melbourne 3086, Australia; Queensland Aphasia Research Centre, University of Queensland, Brisbane 4029, Australia; School of Health and Rehabilitation Sciences, University of Queensland, Brisbane 4072, Australia; NHMRC Centre for Research Excellence in Aphasia Recovery & Rehabilitation, La Trobe University, Melbourne 3086, Australia; Queensland Aphasia Research Centre, University of Queensland, Brisbane 4029, Australia; School of Health and Rehabilitation Sciences, University of Queensland, Brisbane 4072, Australia; NHMRC Centre for Research Excellence in Aphasia Recovery & Rehabilitation, La Trobe University, Melbourne 3086, Australia; Queensland Aphasia Research Centre, University of Queensland, Brisbane 4029, Australia; Queensland Aphasia Research Centre, University of Queensland, Brisbane 4029, Australia; Queensland Aphasia Research Centre, University of Queensland, Brisbane 4029, Australia; School of Health and Rehabilitation Sciences, University of Queensland, Brisbane 4072, Australia; NHMRC Centre for Research Excellence in Aphasia Recovery & Rehabilitation, La Trobe University, Melbourne 3086, Australia; School of Clinical Sciences, Centre for Biomedical Technologies, Queensland University of Technology, Brisbane 4001, Australia; Queensland Aphasia Research Centre, University of Queensland, Brisbane 4029, Australia; School of Health and Rehabilitation Sciences, University of Queensland, Brisbane 4072, Australia; NHMRC Centre for Research Excellence in Aphasia Recovery & Rehabilitation, La Trobe University, Melbourne 3086, Australia; Cognitive Brain Research Unit (CBRU), University of Helsinki, Helsinki 00290, Finland; Centre of Excellence in Music, Mind, Body and Brain, University of Helsinki, Helsinki FI-40014, Finland

**Keywords:** microstructure, corpus callosum, forceps minor, aphasia, stroke

## Abstract

The integrity of the frontal segment of the corpus callosum, forceps minor, is particularly susceptible to age-related degradation and has been associated with cognitive outcomes in both healthy and pathological ageing. The predictive relevance of forceps minor integrity in relation to cognitive outcomes following a stroke remains unexplored. Our goal was to evaluate whether the heterogeneity of forceps minor integrity, assessed early after stroke onset (2–6 weeks), contributes to explaining variance in longitudinal outcomes in post-stroke aphasia. Both word- and sentence-level tasks were employed to assess language comprehension and language production skills in individuals with first-ever left-hemisphere stroke during the early subacute and chronic phases of recovery (*n* = 25). Structural and diffusion neuroimaging data from the early subacute phase were used to quantify stroke lesion load and bilateral forceps minor radial diffusivity. Multiple linear regression models examined whether early subacute radial diffusivity within the forceps minor, along with other factors (stroke lesion load, age, sex and education), explained variance in early subacute performance and longitudinal recovery (i.e. change in behavioural performance). Increased early subacute radial diffusivity in the forceps minor was associated with poor early subacute comprehension (*t* = −2.36, *P* = 0.02) but not production (*P* = 0.35) when controlling for stroke lesion load, age, sex and education. When considering longitudinal recovery, early subacute radial diffusivity in the forceps minor was not linked to changes in performance in either comprehension (*P* = 0.11) or production (*P* = 0.36) under the same control variables. The examination of various language components and processes led to novel insights: (i) language comprehension may be more susceptible to white matter brain health than language production and (ii) the influence of white matter brain health is reflected in early comprehension performance rather than longitudinal changes in comprehension. These results suggest that evaluating baseline callosal integrity is a valuable approach for assessing the risk of impaired language comprehension post-stroke, while also underscoring the importance of nuanced analyses of behavioural outcomes to enhance our understanding of the clinical applicability of baseline brain health measures.

## Teaser

Vadinova *et al*. report that early increased radial diffusivity in forceps minor after stroke explains variance in subacute language comprehension but not production, nor longitudinal recovery. This suggests comprehension is more susceptible to white matter health than production and that baseline white matter health impacts initial performance more than recovery.

## Graphical Abstract

**Graphical Abstract:**
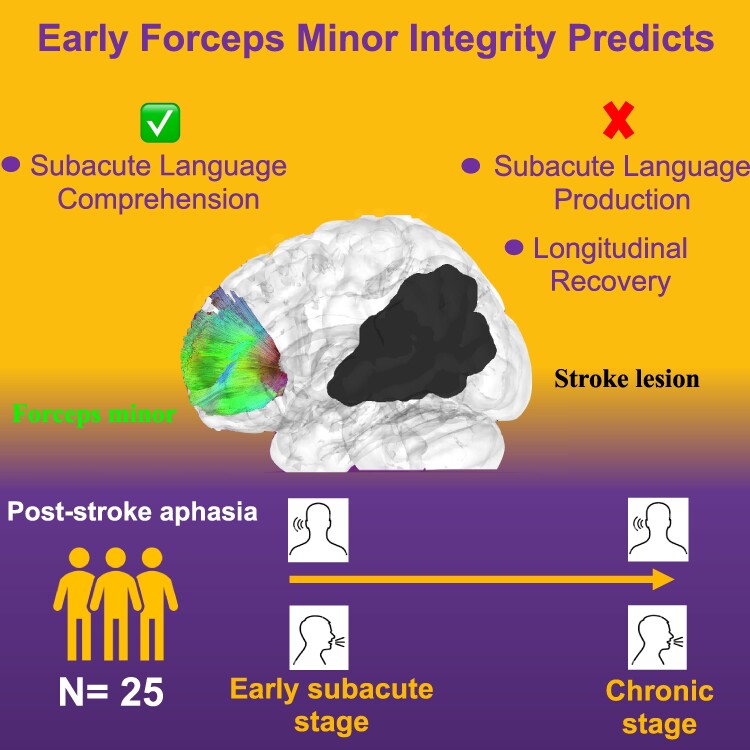

## Introduction

Post-stroke aphasia, a language disorder resulting from focal stroke injury, is characterized by a range of deficits across multiple components of language. While most individuals will experience some recovery of language after a stroke, the degree of recovery varies significantly, and reliable biomarkers of aphasia recovery are yet to be established.^1,2^ Multiple hypotheses have been proposed regarding the precise mechanisms involved in neural recovery. These include upregulation within the preserved language network or upregulation outside the original language network, specifically in the right language homologues and domain-general regions; for a review, see Kiran *et al*.^3^ and Stefaniak *et al*.^4^ This facilitative role of preserved neural structures renders pre-stroke brain health, a potentially relevant factor in influencing recovery trajectories of language after stroke. Several markers of poorer brain health have been proposed in the literature, such as reduced hippocampal volumes,^5^ advanced brain age (as estimated by whole brain machine learning algorithms),^6,7^ the extent of white matter hyperintensities (WMH) lesions^8,9^ and the number of hyperintense vessels (a marker of abnormal haemodynamic function).^10^

The integrity of white matter (WM) microstructure, estimated using diffusion MRI (dMRI), has emerged as a promising candidate biomarker of structural brain health in ageing and disease. The frontal segment of the corpus callosum forceps minor (CC-Fmin) is particularly vulnerable to microstructural deterioration in ageing,^11,12^ as assessed by a variety of dMRI metrics.^13-15^ Different degrees of damage within the CC-Fmin have been linked to the heterogeneity in cognitive abilities in the context of both healthy ageing^13-16^ and in neurodegenerative conditions, e.g. mild cognitive impairment,^17^ Alzheimer's disease,^18,19^ Parkinson's disease^20^ and cerebral amyloid angiopathy.^21^ These apparent associations indicate potential use of CC-Fmin microstructure as a neuroimaging biomarker with cross-diagnostic applicability for predicting functional outcomes.

Within post-stroke aphasia, the relevance of CC-Fmin microstructural degradation to predicting language outcomes is unclear. To the best of our knowledge, three studies have investigated a relationship between distinct diffusion tensor imaging (DTI) metrics of chronic microstructure of several CC segments and chronic aphasia outcomes.^22-24^ Pani *et al*.^24^ revealed an association between reduced entire CC integrity and worse naming and connected speech measures, which was interpreted to reflect post-stroke secondary degeneration. Ivanova *et al*.^23^ examined all callosal segments separately in relation to various chronic language measures and found a positive correlation between higher chronic posterior callosal integrity and better chronic naming outcomes but no other associations. Lastly, Braun *et al*.^22^ found no association between chronic CC-Fmin integrity and overall aphasia severity or response to language therapy in a group of chronic individuals with aphasia (IWA). These findings indicate that chronic measures of callosal integrity may be a useful predictor of impairments across chronic language production skills but not a response to treatment. Notably, all three studies interrogated chronic microstructural measures which conflated age-associated variability in microstructure^12,13,16^ and secondary neurodegeneration triggered by the stroke. On top of the inability to disambiguate the different sources of pathology, understanding the prognostic significance of preserved-tissue biomarkers during the early stages (i.e. acute and early subacute) post-stroke is pivotal, as clinical neuroimaging data are acquired and decisions regarding rehabilitation and ongoing care are usually made during this period. Therefore, it remains to be explored whether a regional averaged microstructure of CC-Fmin, assessed early after stroke onset, may improve longitudinal language prediction models after stroke.

The diffusion tensor model (DTI), the most commonly employed model used to explore changes in WM integrity in ageing, provides several measures, axial diffusivity (Ad), radial diffusivity (RD), mean diffusivity (MD) and fractional anisotropy (FA). Although FA and MD are frequently examined DTI measures,^12,13,16^ given their simultaneous dependence on both Ad and RD, they are prone to inaccuracies in regions of the WM that display non-parallel or opposing patterns of alterations in the underlying Ad and RD.^25,26^ Furthermore, comprehensive analyses with a focus on individual DTI-derived eigenvalues consistently reveal that age-related alterations in composite measures of FA and MD largely arise from increased RD.^27-32^ Tuladhar *et al*.^15^ demonstrated in their extensive analysis of DTI measures and cognitive outcomes that RD in frontal callosal connections accounted for a greater proportion of variance in multiple cognitive measures, including verbal memory, compared to other DTI measures. In the context of post-stroke aphasia, Ivanova *et al*.^23^ observed significantly higher RD values in language-related white matter tracts, including callosal tracts, in IWA compared to controls. Increased RD^30,33,34^ has also been linked to dysmyeliation and demyeliation.

Despite the compelling evidence that pre-stroke brain health contributes to functional recovery after stroke,^5-7,9,10,35^ there is a limited understanding of the utility of various neuroimaging measures of structural brain health. The aim of this study was to determine the extent to which early subacute microstructure of CC-Fmin relates to longitudinal outcomes in post-stroke aphasia. We hypothesized, based on a body of findings in ageing,^13-16^ degenerative disorders^18,19^ and stroke,^23,24^ that a regional averaged RD metric of the CC-Fmin would be associated with aphasia outcomes after stroke, when controlling for stroke lesion volume and other demographic variables, i.e. age, sex and education. Importantly, as different aspects of language can be variably impaired in aphasia (for a detailed discussion, see Wilson *et al*.)^36^ and brain health measures have been shown to differentially relate to the recovery of specific language components,^7,9^ we hypothesized that the RD index of the CC-Fmin (hereafter referred to as RD CC-Fmin) would relate to language comprehension and language production outcomes differently.

## Materials and methods

### Participants

This study retrospectively analysed combined data from two post-stroke aphasia studies, Cohort 1 and Cohort 2. In total, 25 patients were included in the analysis, with 5 participants excluded due to missing data (either dMRI data or education level), and 7 participants excluded due to attrition at the chronic timepoint. The inclusion criteria were (i) a single left-hemisphere stroke, confirmed by neuroimaging, (ii) the presence of aphasia, diagnosed using the Western Aphasia Battery,^37^ (iii) English as a primary language, (iv) availability for an initial assessment at two to six weeks post-stroke onset and (v) able to provide informed consent. The exclusion criteria were (i) history of neurological disorder, mental illness, head trauma, alcoholism or cerebral tumour; (ii) contraindications to MRI; (iii) severity of deficits precluding informed consent, (iv) severe dysarthria or apraxia of speech (determined by a speech pathologist, with apraxia assessed using the Apraxia Battery for adults (ABA-2)^38^ and (v) severe hearing impairment. The study received approval from the University of Queensland Medical Research Ethics Committee and the Queensland Health Human Research Ethics Committee.

### Language assessment

Participants in both cohorts (i.e. Cohort 1 and Cohort 2) completed identical language assessment at the early subacute and chronic stages. Language assessment was performed within 1 week of the neuroimaging data acquisition. For each participant, spoken language comprehension (SpoComp) and spoken language production (SpoProd) performance was measured. The SpoComp score was derived from a combined score of the Auditory Word, Sentence and Paragraph comprehension subtests from the Comprehensive Aphasia Test (CAT).^39^ The SpoProd score was derived by combining scores on the Fluency and Naming (nouns and verbs) subtests from the CAT^39^ with performance on a picture description task^37^ (see Supplementary material for details of the picture description task and analysis). Three IWA were excluded from the SpoProd analysis as their picture description task was not administered at the early subacute stage. Participant information and language scores can be found in Supplementary Table 1.

### Neuroimaging

#### Neuroimaging protocol

Neuroimaging data for both cohorts were acquired only at the early subacute timepoint (mean, 27 days; range, 16–45 days). Data from Cohort 1 (*n* = 10) were collected using Siemens 3 Tesla Trio scanner (Siemens, Erlangen) with a 12-channel head coil. Following data were acquired for each subject: a high-resolution 3D T1-weighted anatomical image [MP-RAGE; TR, 1900 ms; time echo (TE), 2.4 ms; inversion time (TI), 900 ms; (0.9 mm)3 resolution], 2D T2-weighted FLAIR image [TE, 87 ms; TR, 9000 ms; TI, 2500 ms; 36 3-mm slices, 0.9 × 0.9 mm in-plane resolution], a dual-echo diffusion-weighted echo planar imaging (EPI) sequenced dMRI data (TE = 119 ms, TR = 2500 ms, 2.5 × 2.5 × 2.5 mm^3^ resolution, one image with *b* = 0 s/mm^2^, 64 directions at *b* = 3000 s/mm^2^). Data from Cohort 2 (*n* = 15) were collected using a Siemens 3 Tesla MAGNETOM Prisma scanner (Siemens, Erlangen) using a 20-channel head coil. Following data were acquired for each subject: a high-resolution 3D T1-weighted anatomical image [MP2RAGE; TR, 4000 ms; TE, 2.91 ms; TI1, 700 ms; TI2, 2220 ms; (1 mm)^3^ resolution], 3D T2-weighted FLAIR image [TE, 386 ms; TR, 5000 ms; TI, 1800 ms, (1 mm)^3^ resolution], a dual-echo diffusion-weighted EPI sequenced dMRI data (TE = 81 ms, TR = 4000 ms, 2.0 × 2.0 × 2.0 mm^3^ resolution, 11 images with *b* = 0 s/mm^2^, 20 directions at *b* = 1000 s/mm^2^, 60 directions at *b* = 3000 s/mm^2^).

#### dMRI preprocessing and tract reconstruction

Diffusion images were pre-processed using MRTrix (www.mrtrix.org) with FSL (www.fmrib.ox.ac.uk/fsl). Preprocessing included denoising the images and correcting for distortion, eddy currents and motion.^40^ After this, the dMRI data were reconstructed into Montreal Neurological Institute (MNI) space using q-space diffeomorphic reconstruction^41^ that allows for the construction of spin distribution functions in DSI Studio (http://dsi-studio.labsolver.org). During the reconstruction, a mask is used to filter out non-WM structures, increasing the reconstruction efficacy. The b-table was checked by an automatic quality control routine to ensure its accuracy.^42^ Normalization was carried out using the anisotropy map of each participant and a diffusion sampling length ratio of 1.25. Quality of the normalization was inspected using the *R*^2^-values denoting goodness of fit between the participant's anisotropy map and template.

Automatic deterministic fibre tracking was used to track CC-Fmin to calculate the RD index individually for each patient using the DSI Studio (http://dsi-studio.labsolver.org). The automatic tracking uses non-linear registration of subject data to MNI space, followed by seed placement within the atlas tract volume (i.e. in any voxel corresponding to any tract). Each of the generated streamlines is compared to the streamlines associated with each fibre tract from the human connectome project (HCP) tractography atlas^41^ using Hausdorff distances to determine the best match to the streamlines from the target structures in the HCP tractography atlas.^41^ Streamlines matching the target track in the HCP tractography atlas^41^ are retained, and all other streamlines are discarded. The following parameters were used to constrain automatic tracking: a seed limit of 10 000 was applied to obtain an objective measure of tract number, minimum and maximum lengths of 20 and 300 mm were applied (respectively) to disregard tract fragments, and auto track tolerance was set to 20 mm to control the tolerance to spurious fibres. After identification of the tract, the averaged RD index was derived and used in subsequent analyses.

#### Corrected stroke lesion volume calculation

Stroke lesion masks were manually delineated on high-resolution T1-weighted sequences in patient space using MRIcron (https://www.nitrc.org/projects/mricron) by two authors (K.G. and V.V.) and verified by two senior authors (K.M. and S.B.), blinded to behavioural and demographic data. The stroke lesion volume was calculated in native space for subsequent analyses. Corrected stroke lesion volume (SLV) was defined as the ratio of lesion volume to intracranial volume, assessed for each patient prior to normalization.

#### Stroke lesion load CC-Fmin

To confirm that our results were not affected by stroke lesion volume extending into the CC-Fmin, we calculated the stroke lesion load within this tract for each individual. CC-Fmin left segment mask was created in DSI studio software (https://dsi-studio.labsolver.org), based on the HCP1065 atlas.^41^ The proportion of CC-Fmin region of interest (ROI) affected by the stroke lesion was calculated by finding the volume common to both the normalized stroke lesion mask and the CC-Fmin ROI mask (inclusive masking) and then dividing by the total volume of the CC-Fmin ROI mask.

### Statistical analyses

Relationships between the demographic and imaging characteristics were explored using Pearson’s or Spearman’s correlations, as appropriate. As the data in the present study consisted of two separate datasets with distinct acquisition parameters, Levene's Test for Homogeneity of Variance was performed on the RD index data extracted from within CC-Fmin.

We employed multiple linear regression analyses to examine if CC-Fmin microstructure explained performance across longitudinal language outcomes, while accounting for demographic and stroke-related variables. To address the skewed distribution of the corrected SLV variable, a logarithmic transformation was applied prior to analysis. Two outcome measures were defined for each language component as follows: early subacute performance and the change in performance between the early subacute and chronic performances (i.e. delta). The delta for each participant was computed by taking the difference between the raw chronic and early subacute scores for each outcome measure (i.e. delta = chronic performance—early subacute performance). Each outcome language measure served as a separate dependent variable (i.e. early subacute SpoComp, early subacute SpoProd, SpoComp delta, SpoProd delta), resulting in a total of four separate regression analyses.

Each regression analysis included the CC-Fmin RD index (mm^2^/s) and demographic and stroke-related variables, including age (years), sex (male or female), corrected SLV (log) and educational level (UNESCO ISCED: Statistics, 2011). Given the differences in data acquisition between Cohort 1 and Cohort 2, all models included Cohort as a covariate to account for the specific study source. Assumptions of linearity, normality, homoscedasticity and absence of multicollinearity were verified for each model. The chosen level of statistical significance was *α* < 0.05. All analyses were conducted in R (R version 3.0.1, http://www.r-project.org).

## Results

**Figure 1:**
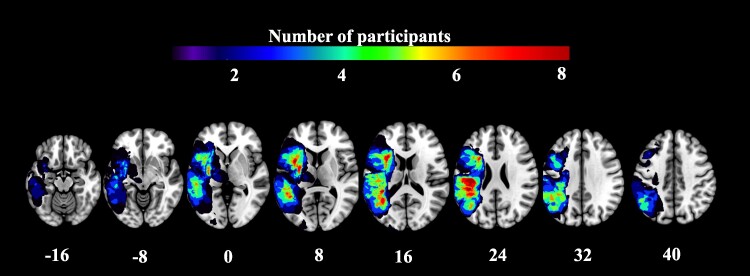
**Lesion topography (*n* = 25).** *Z* plane coordinates (mm) are reported in MNI space.

All demographic and neuroimaging variables at the early subacute stage are reported in Table 1, and an overlay map of all stroke lesions can be found in Fig. 1. There was a moderate positive correlation between the corrected SLV and CC-Fmin RD (*r* = 0.41, *P* = 0.038), indicating that IWA with larger stroke lesions presented with elevated RD within CC-Fmin. There was no difference between the variances of RD index between the two datasets, assessed on Levene's Test for Homogeneity of Variance [*P* = 0.99 (i.e. the homogeneity assumption of the variance was met)]. A two-sample *t*-test indicated that RD was significantly higher in Cohort 1 (mean = 0.61), as opposed to Cohort 2 [mean = 0.48 (*P* < 0.001)]. This difference in means likely reflected differences in acquisition parameters between the cohorts. Cohort 1 had more complex b-shells than Cohort 2. These more complex b-shells model both fast and slower diffusion, and are less prone to model fitting errors due to both the increased design complexity and an increased signal-to-noise ratio due to shorter time echo (TE) times. Education level was significantly higher in Cohort 1 (mean = 4.2), than in Cohort 2 (mean = 2.7; *P* = 0.04). There were no differences between the cohorts in terms of age, sex, corrected SLV and language scores.

**Table 1:** Characteristics: demographic and neuroimaging indices and language outcomes (mean and range)

Characteristic	Entire cohort (*n* = 25) Mean (range)	Cohort 1 (*n* = 10) Mean (range)	Cohort 2 (*n* = 15) Mean (range)
Sex, female/male	8/17	4/6	4/11
Age, years	62 (45–84)	65 (52–84)	61 (45–72)
Education level^a^	3 (0–7)	3 (1–6)	4 (0–7)
Stroke lesion volume, cm^3^	25.07 (1.00–60.49)	14.39 (2.14–56.13)	25.08 (1.00–60.06)
CC-Fmin RD, mm^2^/s	0.56 (0.37–0.74)	0.48 (0.37–0.63)	0.61 (0.52–0.68)
SpoComp early subacute	53.52 (34–66)	56 (37–64)	51.90 (34–66)
SpoComp delta	2.8 (−16–13)	2.5 (−3–13)	3 (−16–11)
SpoProd early subacute	38.07 (2.64–82.86)	43.45 (2.64–82.86)	33.38 (6–64.52)
SpoProd delta	13.39 (−9.01–31.94)	13.93 (−3.91–31.94)	12.94 (−9.01–25.54)

CC-Fmin RD, corpus callosum forceps minor radial diffusivity; SpoComp, spoken comprehension score; SpoProd, spoken production score.

^a^Educational levels scored according to the UNESCO ISCED (International Standard Classification of Education) classification system [Statistics, 2011 (0: early childhood, 1: primary education, 2: lower secondary education, 3: upper secondary education, 4: post-secondary non-tertiary education, 5: short-cycle tertiary education, 6: bachelor's or equivalent, 7: master's or equivalent, 8: doctoral or equivalent).

To confirm that our results were not affected by stroke lesion volume extending into the forceps minor, we calculated the stroke lesion load within the forceps minor. Only two individuals presented with the lesion extending into the CC-Fmin with minuscule lesion loads of 0.05 and 0.06, indicating that the tract was not directly affected by stroke lesion in our patients.

### Model results spoken comprehension

A summary of all the variables and associated statistics at the early subacute stage and the delta can be found in Table 2.

**Table 2:** Summary of multiple regression analyses for early subacute performance and the delta of spoken comprehension and spoken production

	Early subacute SpoComp				Early subacute SpoProd			
	*β*	St error	*t*	*P*	*β*	St error	*t*	*P*
Age	**−0**.**49**	**0**.**12**	**−3**.**82**	**<0**.**001**	−0.52	0.49	−1.06	0.30
Education	0.57	0.61	0.94	0.35	2.62	2.50	1.04	0.31
**CC-Fmin RD, mm^2^/s**	**−47**.**28**	**20**.**03**	**−2**.**36**	**0.02**	−71.13	74.55	−0.96	0.35
**Cohort**	3.30	3.82	0.94	0.39	−1.97	13.24	−0.14	0.88
**Corrected SLV, log**	**−3**.**64**	**1**.**07**	**−3**.**38**	**<0**.**01**	**−9**.**19**	**3**.**80**	**−2**.**41**	**0.02**
**Sex**	**5**.**49**	**2**.**36**	**2**.**32**	**0.03**	13.46	8.05	1.67	0.11

	Delta SpoComp				Delta SpoProd			
	*β*	St error	*t*	*P*	*β*	St error	*T*	*P*
**Age**	**0.35**	**0.10**	**−3.28**	**<0.01**	0.54	0.28	1.89	0.07
**Education**	1.06	0.51	2.08	0.05	−1.12	1.45	−0.77	0.45
**CC-Fmin RD, mm^2^/s**	−27.49	16.84	−1.63	0.11	−40.52	43.29	−0.93	0.36
**Cohort**	−0.54	3.21	−0.17	0.86	−7.05	7.69	−0.91	0.37
**Corrected SLV, log**	**3**.**43**	**0**.**90**	**3**.**79**	**<0**.**01**	1.18	2.21	0.53	0.60
**Sex**	−3.12	1.99	−1.59	0.12	−1.01	4.67	−0.21	0.83

Bold values highlight significant variables.

CC-Fmin RD, corpus callosum forceps minor radial diffusivity; SLV, stroke lesion volume; SpoComp, spoken comprehension score; SpoProd, spoken production score.

### SpoComp scores

The multiple linear regression was significant [*R*^2^ = 0.70, *F*(6, 18) = 10.69 and *P* < 0.001] and indicated that there was a combined significant effect of age (*β* = −0.49, *t* = −3.81 and *P* < 0.01), corrected SLV (*β* = −3.64, *t* = −3.38 and *P* < 0.01), female sex (*β* = 5.49, *t* = 2.32 and *P* = 0.03) and CC-Fmin RD (*β* = −47.28, *t* = −2.36 and *P* = 0.02). An increase of 0.1 mm2/s in CC-Fmin RD was associated with a 4.72 decrease in early subacute SpoComp score (raw).

### SpoComp delta

**Figure 2:**
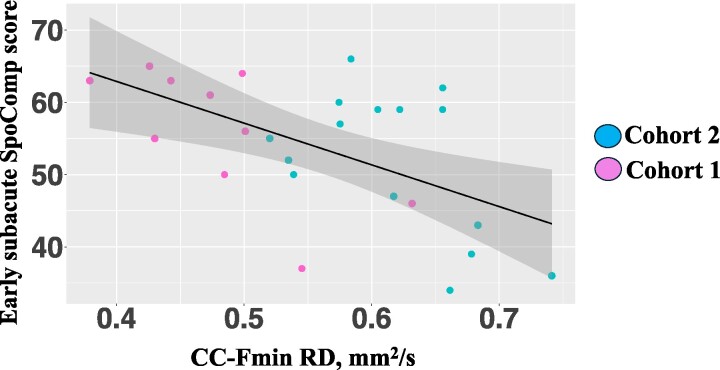
**Multiple linear regression scatterplots: comprehension (*n* = 25).** CC-Fmin RD, corpus callosum forceps minor radial diffusivity; SpoComp, spoken language comprehension. *Test used: simple linear regression, R^2^ = 0.27, F(1, 23) = 10.3, P < 0.01*.

We then explored whether our set of predictors could account for the variance in behavioural recovery, as assessed by the SpoComp measure, achieved by each individual between the early subacute and chronic stages (i.e. SpoComp delta). The multiple linear regression was significant [*R*^2^ = 0.51, *F*(6, 18) = 5.19 and *P* < 0.01] and indicated that there was a significant effect of corrected SLV [*β* = 3.43, *t* = 3.79 and *P* < 0.01] and Age [*β* = 0.35, *t* = 3.28 and *P* < 0.01], indicating that the delta (i.e. behavioural recovery) was larger in individuals with larger initial lesions and more advanced age. RD index within the CC-Fmin did not explain additional variance in the SpoComp delta (Fig. 2).

### Model results spoken production

#### SpoProd score

The multiple linear regression was significant [*R*^2^ = 0.38, *F*(6, 15) = 3.15, *P* = 0.03] and indicated that there was a significant effect of corrected SLV such that individuals with larger lesions scored lower on SpoProd measure at early subacute stage (*β* = −9.19, *t* = −2.41 and *P* = 0.02). No additional variable significantly explained variance in early subacute SpoProd score.

#### SpoComp delta

**Figure 3:**
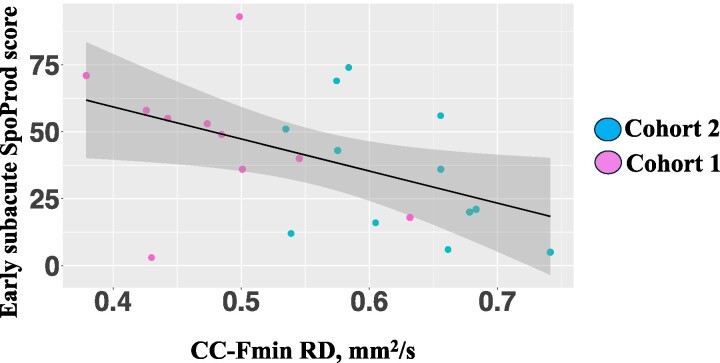
**Multiple linear regression scatterplots: production (*n* = 22).** CC-Fmin RD, corpus callosum forceps minor radial diffusivity; SpoProd, spoken language production. *Test used: simple linear regression, R^2^ = 0.13, F(1, 20) = 4.35, P = 0.49*.

We then explored whether our set of predictors could account for the variance in behavioural recovery, as assessed by the SpoProd measure, achieved by each individual between the early subacute and chronic stages (i.e. SpoProd delta). The multiple linear regression was not significant (*P* = 0.45). None of the investigated predictors explained variance in the SpoProd delta (Fig. 3).

## Discussion

In this study, we probed the impact of the early subacute microstructure of the CC-Fmin on the longitudinal recovery of comprehension and production in post-stroke aphasia. Prior investigations relying on chronic microstructural measures provided equivocal findings regarding the prognostic relationship of chronic callosal microstructure with post-stroke aphasia outcomes.^22-24^ By leveraging early subacute dMRI data (<6 weeks), to minimize the extent of stroke-induced secondary degeneration,^43,44^ this study shows that greater RD in the CC-Fmin is negatively associated with early subacute comprehension outcomes following stroke, while accounting for concurrent stroke and demographic factors. This negative prognostic relationship of elevated RD of frontal callosal microstructure with aspects of language after stroke aligns with the existing evidence on cognitive and functional outcomes in other populations^12-14,17,19,45^ and suggests that characteristics of frontal callosal microstructure may act as a promising biomarker of cognitive outcomes in cohorts comprising both healthy ageing individuals and those affected by neurological conditions.

Interestingly, we found a relationship between frontal callosal microstructure and early subacute SpoComp, but not with early subacute SpoProd scores or the longitudinal recovery of these skills (i.e. delta scores). The discrepant relationship between callosal microstructure over time (i.e. early subacute outcomes, longitudinal recovery) and different components of language (i.e. comprehension, production) underscores the variable contribution that preserved brain tissue may make. We elaborate on the following findings.

### Corpus callosum microstructure and longitudinal language outcomes in post-stroke aphasia

To date, three studies have interrogated the prognostic relationship of chronic region-based CC measures in post-stroke aphasia^22-24^ and yielded discordant findings. Braun *et al*.^22^ failed to observe an association between CC-Fmin microstructure and overall aphasia severity or language treatment gains, while Pani *et al*.^24^ and Ivanova *et al*.^23^ identified a relationship between chronic CC microstructure and chronic production measures, such as naming and speech fluency. Crucially, all above-mentioned studies relied on behavioural and neuroimaging data obtained at various (non-controlled) chronic time points. Chronically acquired neuroimaging data cannot disambiguate the various sources of injury conflated in dMRI data acquired years post-stroke, i.e. deteriorated pre-stroke microstructure^45^ and widespread post-stroke degeneration.^43,44^ Moreover, chronically assessed behavioural performance represents a combination of baseline performance and time-dependent recovery. Our study, distinguished by its longitudinal design with an initial assessment timepoint, was well positioned to dissect initial performance and longitudinal recovery, as well as to investigate the explanatory role of early subacute CC microstructure, serving as a surrogate for pre-stroke values.

Our findings suggest that measures of frontal callosal microstructure are sensitive to processes influencing early subacute comprehension outcomes in post-stroke aphasia. However, we did not observe evidence linking the recovery of language comprehension (i.e. change in performance at a controlled 6-month timepoint) with early subacute CC-Fmin microstructure. Since chronic outcomes amalgamate both baseline performance and longitudinal recovery, CC-Fmin microstructure is associated with chronic comprehension outcomes, yet its contribution is reflected in early performance and not in longitudinal performance changes. This novel discovery underscores the significance of pre-stroke WM brain health measures in cognitive stroke outcomes, highlighting the need for research to employ sensitive measures that effectively dissect the relationship between neuroimaging markers and behaviour in a clinically meaningful manner. A cautious interpretation of our findings suggests that although pre-stroke WM health influences comprehension outcomes post-stroke, its prognostic value for chronic 6-month outcomes (i.e. forecasting the change in performance) may be limited.

A notable discrepancy emerged between the findings of Pani *et al*.^24^ and Ivanova *et al*.,^23^ which linked chronic language production measures with chronic CC microstructure, and our study, which found no impact of early subacute CC-Fmin microstructure on either early subacute production or subsequent recovery. However, these findings are not mutually exclusive, as our study does not extend beyond a 6-month window and cannot address the longitudinal development of either CC-Fmin microstructure and language production outcomes, or their relationship. Nonetheless, collectively, these studies and our research confirm the dynamic and evolving interplay between WM brain health measures and behavioural variables and highlight the inherent limitation of cross-sectional and short longitudinal research designs in accurately capturing their prognostic relationship.

### Individual language components: comprehension and production

The enhanced explanatory power of early subacute frontal callosal microstructure in language comprehension, in contrast to language production, may stem from variations in the upregulation potential within preserved tissue across these language components, and/or the varying contributions of compensatory cognitive skills (e.g. cognitive control, executive function) also reliant on the integrity of preserved brain tissue.

With regard to the first explanation, different language components can be compromised to varying degrees in aphasia, often showing markedly distinct recovery trajectories.^36,46-48^ Therefore, customizing neurobiological and predictive measures for individual language components is important. In a recent large-scale neuroimaging longitudinal study, language comprehension, particularly at a word level, showed the most significant recovery independent of stroke lesion location. This suggests that language comprehension (at least at the word level) might be the most redundantly distributed language component, allowing for high adaptability and compensation within preserved tissue. As a result, the dependence of language comprehension on the health and integrity of preserved brain tissue may be unique compared to other language components.

Second, aphasia is often accompanied by concomitant impairments in verbal and non-verbal cognition.^49-52^ Some evidence suggests that these cognitive deficits may play a significant role in explaining variance in language recovery.^51,53,54^ Given the established link between verbal and non-verbal cognition and reductions in callosal integrity,^13,15,16,19^ our findings might suggest a compensatory role of these additional cognitive abilities in post-stroke language processing. These cognitive contributions may furthermore be specific with regard to different components of language. Behavioural experiments have established an association between language comprehension and executive processing.^55-58^ Plausible anatomical correlates of this behavioural association have been suggested to reside within the bilateral prefrontal cortex (PFC), given that overlapping regions within the PFC have been shown to be engaged during both language comprehension and executive processing tasks in both healthy participants^56,59,60^ and IWA.^61-64^ The CC-Fmin connects several regions within both the medial and lateral portions of the PFC.^65^ As a result, any mediated contribution (functional or neural) of executive processing/cognitive skills to language comprehension is likely to be more susceptible to the cumulative effects of compromised callosal integrity.

### Prognostic relationship of frontal callosal microstructure with cognitive outcomes across cohorts

One of the most prominent and stable findings from research on WM microstructure in ageing is the strength of the prognostic relationship between microstructural properties of frontal callosal connections with cognitive maintenance in healthy ageing and in pathologically ageing cohorts.^13,14,16,19^ The frequently noted decline in integrity of frontal callosal connections during ageing has been attributed to increased susceptibility of frontal WM to vascular risk factors^12^ and naturally lower myelination levels.^14,66^ A novel aspect of this research is the ability of CC-Fmin integrity to explain variance in cognitive outcomes following a stroke. This finding is particularly noteworthy as the adaptive neuroplastic mechanisms involved in compensation following abrupt injury to the brain, likely differ from those involved in healthy ageing or slowly progressing neurodegeneration. Given this, frontal callosal integrity has the potential to be a valuable and clinically meaningful surrogate marker of WM health, relevant to a wide range of compensatory and/or upregulatory processes in ageing and age-associated diseases.

### Limitations

This study has several limitations. Most importantly, this study comprised a small number of participants (*n* = 25), limiting the generalizability of our findings. Incorporating two datasets with varying acquisition parameters could have influenced our ability to identify significant effects of callosal microstructure on individual language components over time. The combination of datasets hinders interpretation of effect sizes of measures such as DTI indices which are known to be sensitive to acquisition parameters. Whilst data merging undeniably induces noise into combined datasets, the concept of combining datasets has emerged as a potential research direction to reduce research waste and harness the availability of smaller existing datasets.^67^ Furthermore, the HCP atlas was selected because it represents the largest expert-vetted population-averaged white matter atlas, providing high accuracy by capturing averaged white matter anatomy. However, it is important to note that this atlas is based on a healthy adult population and not specifically on older individuals with elevated cardiovascular risk profiles, which may have impacted the tracking accuracy of CC-Fmin.

Second, whilst we have minimized the extent of secondary degeneration by interrogating data acquired within several weeks of stroke onset (<6), we cannot exclude the possibility that some stroke-related degeneration has occurred within our data. Correlational analyses indicated a weak negative correlation between early subacute corrected SLV and early subacute CC-Fmin RD in patients who returned for chronic assessment. This moderate association can be explained by several factors. Firstly, emerging stroke-related degeneration may have occurred within the timeline of the early subacute stage. Second, individuals with more significant age-related white matter degradation and poorer brain health might be more prone to larger, more severe strokes. To disentangle these, hyperacute or acute dMRI data are necessary. Lastly, temporal variability of up to 1 week between neuroimaging and behavioural data acquisitions may have introduced some noise into our data. Although temporal discrepancies are more critical during the hyperacute and acute stages, where behavioural performance undergoes dynamic and rapid changes, some unwanted variability may have affected the precision of the identified relationship between microstructural and behavioural measures.

### Future research

The importance of pre-stroke WM microstructure in predicting aphasia outcomes is a nascent area of research and further investigations are required to (i) gain insights into the mechanisms by which neuroplastic recovery processes may be disrupted in patients with severely compromised pre-stroke microstructure and (ii) explore the longitudinal interaction of compromised microstructure at stroke onset and well-documented secondary post-stroke degeneration.

Our findings, in conjunction with previous investigations of frontal callosal microstructure,^13,16,17,45^ are unable to determine the mechanisms responsible for observed relationships between CC-Fmin microstructure and cognitive outcomes. Multiple candidate mechanisms, not mutually exclusive, can be considered. These include the potential involvement of frontal callosal connections in networks crucial for diverse cognitive outcomes and the hypothesis that the integrity within these connections serves as a sensitive indicator of overall brain health, influencing cognitive outcomes in turn. To test between these hypotheses, converging functional network-level analyses are imperative to provide more definitive mechanistic insights into suboptimal stroke recovery in the presence of degraded callosal microstructure.

Second, our research established a significant variability in frontal callosal microstructure early after stroke onset. Given that focal stroke lesions trigger widespread progressive microstructural degeneration,^43,44^ it remains inconclusive as to whether the two processes (i.e. pre-stroke compromised microstructure, post-stroke degeneration) interact and whether that interaction incurs further behavioural consequences. Hypothetically, patients with severely compromised pre-stroke microstructure may be more vulnerable to stroke-related secondary degeneration and hence exacerbated long-term aphasia severity.

## Conclusion

Here, we offer the first investigation into the prognostic relationship between the early subacute microstructure of the frontal CC, a region shown to be especially vulnerable to age-related degradation,^11,12^ and long-term language outcomes after a stroke. Although early CC integrity is seldom considered in aphasia research and the assessment of language network WM integrity is the most prevalent assessment method,^68-70^ our findings argue in favour of considering tracts outside the core language network, such as the CC-Fmin, when attempting to explain the variability in outcomes in post-stroke aphasia. Unlike previous research on brain health measures in post-stroke aphasia,^5-7,9,10,35^ we undertook detailed analyses of various language components (i.e. language comprehension and language production) and processes (i.e. early subacute performance and longitudinal recovery). This approach enabled us to yield the novel finding that early subacute CC-Fmin microstructure may be more sensitive to processes that impact language comprehension outcomes than those affecting language production outcomes. Crucially, the finding that lower CC-Fmin microstructure is associated with early subacute language comprehension, but not with the longitudinal recovery of comprehension, extends our understanding of the potential prognostic utility of baseline brain health measures in a clinical setting. Consequently, our findings provide a strong rationale for conducting longitudinal stroke investigations with multiple controlled chronic time points, specifically focusing on modelling baseline performance and behavioural recovery separately.

## Supplementary Material

fcae370_Supplementary_Data

## Data Availability

The data that support the findings of this study are available from the corresponding author, V.V., upon reasonable request.
