# Supplementary material for: Early subacute frontal callosal microstructure and language outcomes after stroke
Source: Brain Commun. 2025 Jan 21;7(1):fcae370. doi: 10.1093/braincomms/fcae370 (PMC11753390; doi:10.1093/braincomms/fcae370)
Supplement: fcae370_Supplementary_Data [file fcae370_supplementary_data.docx]

**SUPPLEMENTARY MATERIAL**

**Early subacute frontal callosal microstructure and language outcomes after stroke**

Veronika Vadinova^1,2,3^, Sonia E. Brownsett^1,2,3^, Kimberley L. Garden^1,2,3^, Tracy Roxbury^1^, Katherine O’Brien^1^, David A. Copland ^1,2,3^, Katie L. McMahon^6^, Aleksi J. Sihvonen^1,2,3,4,5^

1 Queensland Aphasia Research Centre, University of Queensland, Brisbane, Australia

2 School of Health and Rehabilitation Sciences, University of Queensland, Brisbane, Australia

3 Centre of Research Excellence in Aphasia Recovery and Rehabilitation, La Trobe University, Melbourne, Australia

4 Cognitive Brain Research Unit (CBRU), University of Helsinki, Helsinki, Finland

5 Centre of Excellence in Music, Mind, Body and Brain, University of Helsinki, Helsinki, Finland

6 School of Clinical Sciences, Centre for Biomedical Technologies, Queensland

University of Technology, Brisbane, Australia

**Corresponding author:**

Veronika Vadinova

Queensland Aphasia Research Centre, University of Queensland, Australia

St Lucia 4072

Brisbane, Australia

veronika.vadinov@gmail.com

+61 0490916670

**1. Calculation of Spoken Production score.**

*Fluency, Naming assessments*

All raw scores from the Comprehensive Aphasia Test (CAT) subtests were independently scored by two qualified speech pathologists blinded to neurological data.

*Picture description*

Transcriptions of picture descriptions from the Western Aphasia Battery (WAB) (ref) were coded by two researchers blinded to neurological, demographic and timepoint data according to the procedures in the Codes for the Human Analysis of Transcripts (CHAT) (<https://talkbank.org/>). These coded transcriptions were then analysed using the Computerized Language Analysis (CLAN) (https://www.talkbank.org/) software, which enables automatic computation of various linguistic indices. We derived three indices to capture the lexical (informativeness), grammatical (*grammatical wellformedness)* and fluency (speed) properties of language produced using this stimulus*.* Speed (number of intelligible words per minute) and grammatical wellformedness (percentage of grammatically correct utterances produced, excluding empty speech, jargon and irrelevant content) were automatically calculated by the CLAN software. Informativeness was assessed by calculating the number of information carrying units (ICU). ICU's were identified separately on uncoded transcripts by two researchers blinded to neurological data, including a speech pathologist. Any disagreements in ICU identified were resolved by consensus. An informativeness index was calculated as the number of accurate ICU's minus all inappropriate ICUs (such as; phonological paraphasias, semantic paraphasias, neologisms, circumlocutions) per minute. Final SpoProd measure was calculated as follows:

$SpoProd score = \frac{fluency + naming + speed + grammatical wellformedness + informativeness}{5}$

**Supplementary Table 1. Neuroimaging, socio-demographic and behaviour characteristics of included participants**

| **ID** | **Group** | **Age** | **Gender** | **Education** | **Raw lesion volume** | **Corrected lesion volume** | **Fmin RD** | **SpoProd early subacute** | **SpoComp early subacute** | **SpoComp delta** | **SpoProd delta** |
| --- | --- | --- | --- | --- | --- | --- | --- | --- | --- | --- | --- |
| PAPAR_013 | PA | 65 | M | 4 | 57.14 | 3.09 | 0.66 | 6.00 | 34.00 | 11.00 | 25.54 |
| PAPAR_018 | PA | 54 | M | 4 | 49.58 | 3.18 | 0.52 | NA | 55.00 | 9.00 | NA |
| PAPAR_019 | PA | 58 | M | 4 | 60.06 | 3.26 | 0.62 | NA | 47.00 | 5.00 | NA |
| PAPAR_020 | PA | 45 | M | 0 | 17.21 | 1.09 | 0.62 | NA | 59.00 | -7.00 | NA |
| PAPAR_022 | PA | 55 | F | 4 | 6.51 | 0.39 | 0.66 | 32.10 | 62.00 | -16.00 | 17.36 |
| PAPAR_023 | PA | 70 | M | 4 | 28.83 | 1.70 | 0.54 | 4.78 | 50.00 | 7.00 | 23.36 |
| PAPAR_024 | PA | 61 | F | 6 | 6.41 | 0.44 | 0.58 | 48.16 | 57.00 | 5.00 | 14.90 |
| PAPAR_029 | PA | 64 | F | 6 | 17.63 | 1.05 | 0.66 | 52.59 | 59.00 | 5.00 | 21.97 |
| PAPAR_032 | PA | 70 | M | 1 | 28.89 | 1.77 | 0.53 | 47.51 | 52.00 | 3.00 | 21.28 |
| PAPAR_033 | PA | 72 | M | 4 | 60.50 | 3.43 | 0.74 | 10.63 | 36.00 | 5.00 | 3.91 |
| PAPAR_034 | PA | 65 | M | 4 | 1.01 | 0.06 | 0.57 | 64.40 | 60.00 | -2.00 | 10.03 |
| PAPAR_037 | PA | 66 | M | 6 | 48.25 | 3.23 | 0.68 | 16.15 | 43.00 | 11.00 | 15.11 |
| PAPAR_040 | PA | 68 | M | 6 | 20.99 | 1.44 | 0.68 | 27.82 | 39.00 | 10.00 | 9.84 |
| PAPAR_042 | PA | 52 | M | 4 | 24.93 | 1.58 | 0.60 | 28.31 | 59.00 | -1.00 | 1.04 |
| PAPAR_056 | PA | 51 | F | 7 | 55.11 | 3.85 | 0.58 | 64.52 | 66.00 | 0.00 | -9.02 |
| TR_01 | TR | 81 | F | 2 | 15.70 | 1.06 | 0.55 | 37.23 | 37.00 | 13.00 | 14.94 |
| TR_02 | TR | 49 | M | 2 | 6.59 | 0.34 | 0.43 | 50.99 | 65.00 | -1.00 | 7.41 |
| TR_06 | TR | 56 | M | 2 | 21.47 | 1.29 | 0.43 | 2.64 | 55.00 | 6.00 | 31.95 |
| TR_07 | TR | 84 | F | 1 | 14.46 | 0.93 | 0.63 | 15.71 | 46.00 | 0.00 | 18.74 |
| TR_10 | TR | 70 | F | 1 | 2.81 | 0.19 | 0.44 | 52.75 | 63.00 | -3.00 | 14.97 |
| TR_12 | TR | 76 | M | 6 | 6.40 | 0.40 | 0.50 | 35.29 | 56.00 | 4.00 | 15.72 |
| TR_13 | TR | 52 | M | 4 | 6.38 | 0.37 | 0.38 | 66.07 | 63.00 | 2.00 | 1.17 |
| TR_14 | TR | 63 | F | 1 | 56.14 | 3.09 | 0.48 | 45.36 | 50.00 | 5.00 | 13.05 |
| TR_16 | TR | 54 | M | 4 | 2.15 | 0.14 | 0.50 | 82.86 | 64.00 | -2.00 | -3.91 |
| TR_17 | TR | 66 | M | 4 | 11.84 | 0.75 | 0.47 | 45.66 | 61.00 | 1.00 | 25.33 |
